# Postharvest melatonin treatment inhibited longan (*Dimocarpus* *longan* Lour.) pericarp browning by increasing ROS scavenging ability and protecting cytomembrane integrity

**DOI:** 10.1002/fsn3.2448

**Published:** 2021-07-05

**Authors:** Tao Luo, Feilong Yin, Lingyan Liao, Yunfen Liu, Boyang Guan, Min Wang, Tingting Lai, Zhenxian Wu, Liang Shuai

**Affiliations:** ^1^ College of Horticulture South China Agricultural University/Guangdong Provincial Key Laboratory of Postharvest Science of Fruits and Vegetables/Engineering Research Center of Southern Horticultural Products Preservation, Ministry of Education Guangzhou China; ^2^ College of Food and Biological Engineering/Institute of Food Science and Engineering Technology Hezhou University Hezhou China; ^3^ Key Laboratory of Biology and Genetic Improvement of Horticultural Crops (South China), Ministry of Agriculture and Rural Affairs/Guangdong Litchi Engineering Research Center Guangzhou China

**Keywords:** antioxidants, Longan (*Dimocarpus* *longan* Lour.) fruit, melatonin treatment, oxygen free radical (ROS), pericarp browning, ROS scavenging enzymes

## Abstract

Postharvest melatonin treatments have been reported to improve the quality and storability, especially to inhibit browning in many fruits, but the effect had not been systematically investigated on longan fruit. In this study, the effect of 0.4 mM melatonin (MLT) dipping on the quality and pericarp browning of longan fruits stored at low temperature was investigated. The MLT treatment did not influence the TSS content of longan fruits but lead to increased lightness and h° value while decreased a* value of pericarp. More importantly, the treatment significantly delayed the increase in electrolyte leakage and malonaldehyde accumulation, inhibited the activities of polyphenol oxidase and peroxidase, and thus retarded pericarp browning. In addition, the treatment significantly inhibited the production of O_2_^•−^ and H_2_O_2_ while promoted the accumulation of glutathione, flavonoids, and phenolics at earlier storage stages in longan pericarp. Interestingly, the activities of ascorbate peroxidase (APX) and superoxide dismutase (SOD) were significantly upregulated but activities of catalase were downregulated in the MLT‐treated longan pericarp. MLT treatment effectively enhanced APX and SOD activities, increased flavonoid, phenolics, and glutathione content, protected cytomembrane integrity, inhibited the production of O_2_^•−^ and H_2_O_2_ and browning‐related enzymes, and thus delayed the longan pericarp browning.

## Graphical abstract

Our results showed that 0.4 mM melatonin (MLT) dipping significantly increased the ROS scavenging ability by enhancing the activities of SOD and APX as well as increasing the content of GSH, phenolics, and flavonoids (especially at the earlier storage stages). This might result in the decreased level of O_2_^•−^ and H_2_O_2_ and lower MDA (membrane lipid oxidation products) content, and thus, the treatment protected the cytomembrane integrity and prevented the pericarp from enzymatic browning by PPO and POD. In conclusion, postharvest melatonin treatment inhibited longan pericarp browning by increasing ROS scavenging ability and protecting cytomembrane integrity.

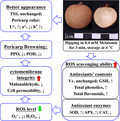

## 1 INTRODUCTION

Longan (*Dimocarpus longan* Lour.), one of the typical tropical and subtropical fruits with high commercial value, is a kind of globally consumed fruit but is extremely unresistant to long‐term postharvest storage and transportation (Han et al., 2020; Luo, Niu, et al., 2019; Luo et al., 2021; Luo, Shuai, et al., 2019). Longan fruits are usually harvested in summer with high temperatures and humidity. Therefore, deterioration of longan fruit characterized as pericarp browning and aril breakdown quickly occurs within a few days without low‐temperature storage (Han et al., 2020; Luo, Niu, et al., 2019). Compared with the aril breakdown of longan fruits, which was an indicator of interior quality deterioration and significantly varied among cultivars, the pericarp browning is a much more common appearance index of senescence in longan pericarp (Jiang et al., 2002). Postharvest pericarp browning of longan was proved to be related to the oxidation of peroxidase (POD) and polyphenol oxidase (PPO) on phenolics (Jiang et al., 2002). This oxidation process might be exacerbated by the burst of reactive oxygen species (Lin et al., 2014), hydrogen peroxide‐induced energy deficiency (Lin, Lin, Chen, et al., 2016), and peroxidation of membrane lipid (Lin, Lin, Lin, et al., 2016), all of which might be caused by the absence of enzymatic and nonenzymatic ability for scavenging free radicals. In addition, destroying of cellular compartmentation and integrity of cell membrane, which are possibly induced by water loss (Lin et al., 2010), pathogen infection (Chen et al., 2014), and other stresses, are also important factors promoting postharvest browning of longan pericarp.

In the past three decades, techniques including chemical, physical, and biological treatments have been developed to control postharvest loss, reduce browning, and extend the storage life of longan fruit (Han et al., 2020; Jiang et al., 2002; Luo, Niu, et al., 2019). Traditional chemical treatments such as SO_2_ fumigation (or other sulfur treatments) were widely used for long‐period prevention of pericarp browning in actual longan production due to their powerful inhibition of PPO (Chitbanchong et al., 2009; Hai et al., 2011; Han et al., 2020; Jiang et al., 2002). Similarly, fumigation with chlorine dioxide (or sodium chlorite as donor) was able to significantly reduce postharvest loss of longan fruits (Chumyam et al., 2021; Intarasit et al., 2018; Khunpon et al., 2011; Saengnil et al., 2014; Vichaiya et al., 2020). Chemicals including hydrochloric acid (Apai et al., 2010), propyl gallate (Lin et al., 2013), 2‐butanol (Li et al., 2015), and α‐aminoisobutyric acid or β‐aminoisobutyric acid (Wang et al., 2015) were reported to be effective to control postharvest loss and pericarp browning of longan fruits. Moreover, chitosan (Jiang et al., 2001) or chitosan containing citric acid (or citric acid plus potassium sorbate) for coating (Apai et al., 2009), sealing films (Khan et al., 2016), controlled atmospheres (Tian et al., 2002), pure oxygen (Su et al., 2005), ozone in combination with some organic acids (Whangchai et al., 2006), and sodium nitroprusside (donor of NO) (Duan et al., 2007) were also proved as an effective method for reducing pericarp browning and postharvest loss of longan. However, SO_2_ fumigation and fungicide treatments under residue limits were still widely used for retarding deterioration of longan fruits due to their long effective action and convenience.

Melatonin (MLT), namely N‐acetyl‐5‐methoxytryptamine, was proved to be effective to keep the postharvest quality and health properties of fresh fruit. The documents about its application on postharvest fruit concerning the shelf‐life extension and quality maintenance increased rapidly in the past decades (Yun et al., 2021). The delaying of postharvest MLT treatment on the senescence was reported on many fruits such as apple (Onik et al., 2020), citrus (Lin et al., 2019), grape (Wang et al., 2020), kiwifruit (Hu et al., 2018), mango (Rastegar et al., 2020), peach (Gao et al., 2016), pear (Liu et al., 2019), strawberry (Liu et al., 2018), sweet cherry (Xia et al., 2020), tomato (Li et al., 2019), and litchi fruit (Zhang et al., 2018). The decreased physiological characters in MLT‐treated fruit compared with control fruit were malonaldehyde (MDA) or/and electrolyte leakage, which were reported in apple (Onik et al., 2020), kiwifruit (Hu et al., 2018), peach (Gao et al., 2016), strawberry (Liu et al., 2018), sweet cherry (Xia et al., 2020), tomato (Li et al., 2019), pomegranate (Jannatizadeh et al., 2019), and litchi fruit (Zhang et al., 2018). More importantly, postharvest MLT treatment resulted in significant changes in the ROS level, the content of antioxidants, and the enzymatic abilities for scavenging free radicals. Most of the literature reported that MLT treatment leads to increased content of antioxidants such as ascorbic acid, flavonoids, phenolics, and anthocyanins but decreased activity of PPO. Therefore, the postharvest MLT treatment was proved to significantly retard the browning of pomegranate (Jannatizadeh et al., 2019), sweet cherry (Xia et al., 2020), and litchi fruit (Zhang et al., 2018). However, the effect of MLT treatment on the postharvest quality and deterioration of longan fruits have not been investigated yet. In this study, the “Chuliang” longan fruits were treated with prochloraz to eliminate the influence of microorganisms on the deterioration. Then, the longan fruits were treated with dipping in 0.4 mM MLT and stored at low temperature. The contents of total soluble solids (TSS), pericarp chromatic values, browning index, membrane permeability index (MDA and electrolyte leakage), browning‐related enzyme activities, the content of ROS and antioxidants, and activities of enzymes for scavenging free radicals were determined to evaluate the effect of MLT on the deterioration especially pericarp browning of longan fruits. This study was expected to clarify the mechanism for inhibition of MLT on longan pericarp browning and thus provide a theoretical basis for applying MLT in developing new preservative methods.

## 2 MATERIALS AND METHODS

### 2.1 Longan fruits, treatments, and storage

Commercial mature “Chuliang” longan fruits were harvested in an orchard at the Hezhou Academy of Agricultural Sciences (Guangxi province, China) and immediately transported to the laboratory. More than 1,200 fruits with no disease and no damage were selected and dipped in 500 mg/L prochloraz solution for 3 min. The fruits were then dried at room temperature for 30 min. After that, 600 fruits were dipped in 0.4 mM MLT (98% HPLC purity, Macklin, Shanghai Macklin Biochemical Co., Ltd) for 3 min (abbreviated to 0.4 mM MLT), and the other 600 fruits were dipped in clean water for 3 min (CK). The fruits were naturally dried for 60 min, and every 20 fruits were packed into one polyethylene bag (0.03 mm thick). The fruits were then stored at 4 ± 1°C and 85% relative humidity.

### 2.2 Determination of chromatic value

The fruit color was measured, respectively, at 0, 8, 16, 24, and 32 days after storage (DAS) using a color analyzer (KONICA MINOLTA CR‐300). Ten fruits were randomly selected and the L*, a*, b*, c*, and h° values on equatorial plane of each fruit were subjected to three detections. The color index (CI) was calculated by the equation 1 according to Luo et al. (2015):

(1) CI=1000a/Lb

### 2.3 Determination of pericarp browning index

Browning index of inner pericarp was calculated based our reported method (Luo, Niu, et al., 2019). The extent of the total browned area (TBNA) on each pericarp was estimated using the following scales: score =1, no browning; score =2, TBNA was 0%–25%; score =3, TBNA was 25.1%–50%; score =4, TBNA was 50.1%–75%; score =5, TBNA was 75.1%–99.9%; score =6, TBNA was 100%. The pericarp browning index of 30 fruits at each time point was calculated according to the Equation 2:

(2) $$\text{Pericarp browning index}=\left(\sum \text{TBNA of each fruit}\right)/30$$

### 2.4 Sampling and determination of TSS content

The longan fruits were, respectively, sampled at 0, 8, 16, 24, and 32 DAS. The pericarp and aril of each longan fruit were separated. The collected pericarp of treated or CK fruits was immediately frozen in liquid nitrogen, ground and stored at −80°C until be used. The separated aril from each fruit was used for juicing and determination of TSS content by a brix refractometer (PR‐32α, ATAGO). Sampling and TSS analysis were performed by three times (one bag per time).

### 2.5 Analysis of pericarp relative electrolytic leakage

The cell membrane permeability of pericarp was measured according to a previously reported method (Zhang et al., 2018).

### 2.6 Determination of MDA content in pericarp

The MDA content was determined using the thiobarbituric acid (TBA) method with some adjustments (Zhang et al., 2018). A 1.0 g sample of powder was fully mixed with 8 ml precooled 10% TCA solution and shaken for 30 s. The sample was extracted for 10 min in an ice‐bath. After a centrifugation for 20 min at 5,000 × *g* and 4°C, 2 ml supernatant was moved out to be mixed with 2 ml TBA and then heated in boiling water for 20 min. After be cooled and centrifuged at 5,000 × *g* for 5 min, the absorbance of supernatant at 532 nm was recorded and the value for nonspecific absorption at 600 nm and 450 nm was subtracted. Mixture of 2 ml 10% TCA and 2 ml TBA was set as a blank sample. All samples were subjected to three repeats.

### 2.7 Determination of polyphenol oxidase (PPO) and peroxidase (POD) enzymatic activities

Polyphenol oxidase activity was determined according to Zhang et al. (2018) with some modification. A 1.0 g sample of powder was added into a precooled tube containing 8 ml 50 mM PBS (pH 5.5) and 0.2 g PVP, and then fully mixed. After a centrifugation at 12,000* × g* and 4°C for 20 min, the supernatant was removed to a new tube. The precipitate was washed by another 6 ml 50 mM PBS (pH 5.5) and centrifuged at 12,000 × *g* and 4°C for 20 min. The supernatants of two centrifugations were merged into one tube and the volume was filled to constant 20 ml by deionized water. The reaction system containing 0.1 ml enzymatic extraction, 3.9 ml 10 mM acetic acid buffer (pH 5.5), and 1 ml 0.1 M catechol was kept at 35°C for 10 min. After that, the solution was cooled by ice‐bath and mixed with 2 ml 30% TCA to stop the reaction. The enzymatic extraction inactivated by boiling water was set as a control sample. The increase of 0.01 OD_525nm_ per minute was recorded as one enzyme activity unit (U). The result was expressed as U g^‐1^ FW.

The activity of POD in pericarp was assayed by a change of absorbance at 470 nm caused by production of tetraguaiacol from guaiacol in the presence of H_2_O_2_ (Zhang et al., 2018). A change of 0.01 in the absorbance per minute was recorded as one unit of POD enzyme activity. The result was expressed as U g^‐1^ FW.

### 2.8 Measurement of superoxide anion (O_2_^•−^) and H_2_O_2_ contents in pericarp

The O_2_^• −^ production rate was determined according to the procedure described in Lin et al. (2014) and Zhang et al. (2018) with slight modifications. Briefly, 1 g homogenized pericarp powder was fully mixed with 8 ml of 50 mM pH 7.8 phosphate buffer containing 1 mM ethylene diamine tetraacetic acid, 1% polyvinylpyrrolidone (w/v), and 0.3% Triton X‐100. After a shaking for 30 s, the sample was extracted for 10 min in ice. After a centrifugation for 15 min at 10,000 × *g* and 4°C, the supernatant was used for the determination of the O_2_^•−^ production rate. The reaction systems (0.5 ml supernatant, 0.5 ml deionized water, 1 ml 50 mM PBS [pH 7.8] and 1 ml 1 mM hydroxylamine hydrochloride) were fully mixed and incubated at 25°C for 1 hr. One milliliter of 17 mM *p*‐Aminobenzene sulfonic acid and 1 ml α‐naphthylamine (7 mM) were added and incubated at 25°C for 20 min. The absorbance at 530 nm was recorded. A standard curve with sodium nitrite was built. The O_2_^•−^ production rate was calculated from the reaction equation of O_2_^•−^ with hydroxylamine and expressed as μmol/g FW min^−1^.

The H_2_O_2_ content was measured according to the method described by Zhang et al. (2018) with some modifications. Powdered pericarp samples (1 g) were homogenized with 8 ml precooled acetone. After a shaking for 30 s, the sample was extracted for 10 min in an ice‐bath. The sample was centrifuged at 10, 000 × *g* and 4°C for 20 min. Then, 1 ml of supernatant was mixed with 0.1 ml 10% Ti(SO_4_)_2_ (v/v, dissolved in concentrated HCl) and 0.2 ml of concentrated NH_3_H_2_O, and the reaction was kept for 5 min. After be centrifuged at 10,000 × *g* and 4°C for 20 min, the residue was washed three times with precooled acetone. The precipitate without pigments were dissolved in 3 ml 2 M H_2_SO_4_. After be centrifuged at 5,000 × *g* and 4°C for 15 min, the absorbance of the supernatant at 412 nm was recorded. The H_2_O_2_ content was calculated according to a H_2_O_2_ standard curve and expressed as μmol/g FW.

### 2.9 Measurement of contents of ascorbic acid and glutathione (GSH) in pericarp

The supernatant for determining the content of ascorbic acid and GSH was extracted according to the same process used for measurement of MDA. One milliliter supernatant was mixed with 1 ml 5% TCA and 1 ml ethanol. After a full mixture by shaking, 0.5 ml 0.4% phosphoric acid ethanol buffer, 1 ml 0.5% bathophenanthroline‐ethanol, and 0.5 ml 0.03% FeCl_3_‐ethanol were added and the reaction were kept at 30°C for 90 min. The absorbance of the solution at 543 nm was recorded. The ascorbic acid content was calculated according to a standard curve and expressed as μmol/g FW.

For measuring GSH content, 0.5 ml supernatant was mixed with 2 ml 0.1 M PBS buffer (pH 7.8) and 0.5 ml 5,5'‐Dithiobis‐2‐nitrobenzoic acid (4 mM). The solution was kept at 30°C for 20 min. The absorbance of the solution at 412 nm was recorded. The GSH content was determined according to a standard curve and expressed as μmol/g FW.

### 2.10 Detection of the total flavonoid and total phenolic acid contents in pericarp

The extraction for analyzing total phenolics and total flavonoid content was same to the previous method (Luo, Niu, et al., 2019). The absorbance of extract was firstly assayed at 325 nm for flavonoid determination and then at 280 nm for determining content of total phenolics. The total phenolics and total flavonoid content were calculated and expressed as OD_325nm_ g^‐1^ FW or OD_280nm_ g^‐1^ FW.

### 2.11 Assays of superoxide dismutase (SOD), ascorbate peroxidase (APX), and catalase (CAT) activities

One gram pericarp powder was fully mixed with 8 ml precooled 0.1 M phosphate buffer (pH 7.8, containing 5 mM DTT and 2% PVP, w/v) by shaking for 30 s. The sample was extracted for 10 min in an ice‐bath. After be centrifuged at 8,000 × *g* and 4°C for 15 min, the supernatant was used for enzymatic activity measurement of SOD (Zhang et al., 2018) and CAT (Lin et al., 2014). SOD activity was measured by detecting the capacity of the supernatant to inhibit the photoreduction of nitroblue tetrazolium. The amount of enzyme resulting in 50% inhibition of NBT reduction at 560 nm was regarded as 1 unit (U) of SOD activity (Zhang et al., 2018). CAT activity decomposing H_2_O_2_ was assayed by monitoring the change of absorbance at 240 nm. The capacity causing a change of 0.01 of the absorbance per minute was recorded as 1 unit (U) of CAT activity (Lin et al., 2014).

One gram pericarp powder was shaken for 30 s and fully mixed with 8 ml precooled 0.1 M potassium phosphate buffer (pH 7.5, 1 mM EDTA, 2% PVP, w/v, and 1 mM ascorbic acid). The sample was extracted for 10 min in an ice‐bath. After be centrifuged at 8,000 × *g* and 4°C for 15 min, the supernatant was removed to measure APX activity. The capacity resulting in a change of 0.01 of the absorbance at 290 nm per minute was regarded as 1 unit (U) of APX activity (Lin et al., 2014).

### 2.12 Statistical analysis

The variance of data was determined by SPSS software package release 17.0 (SPSS Inc.). Multiple comparisons were performed by one‐way ANOVA based on Duncan's multiple range tests.

## 3 RESULTS

### 3.1 Effect of MLT treatment on the TSS content and exterior quality of low‐temperature stored longan fruits

**FIGURE 1:**
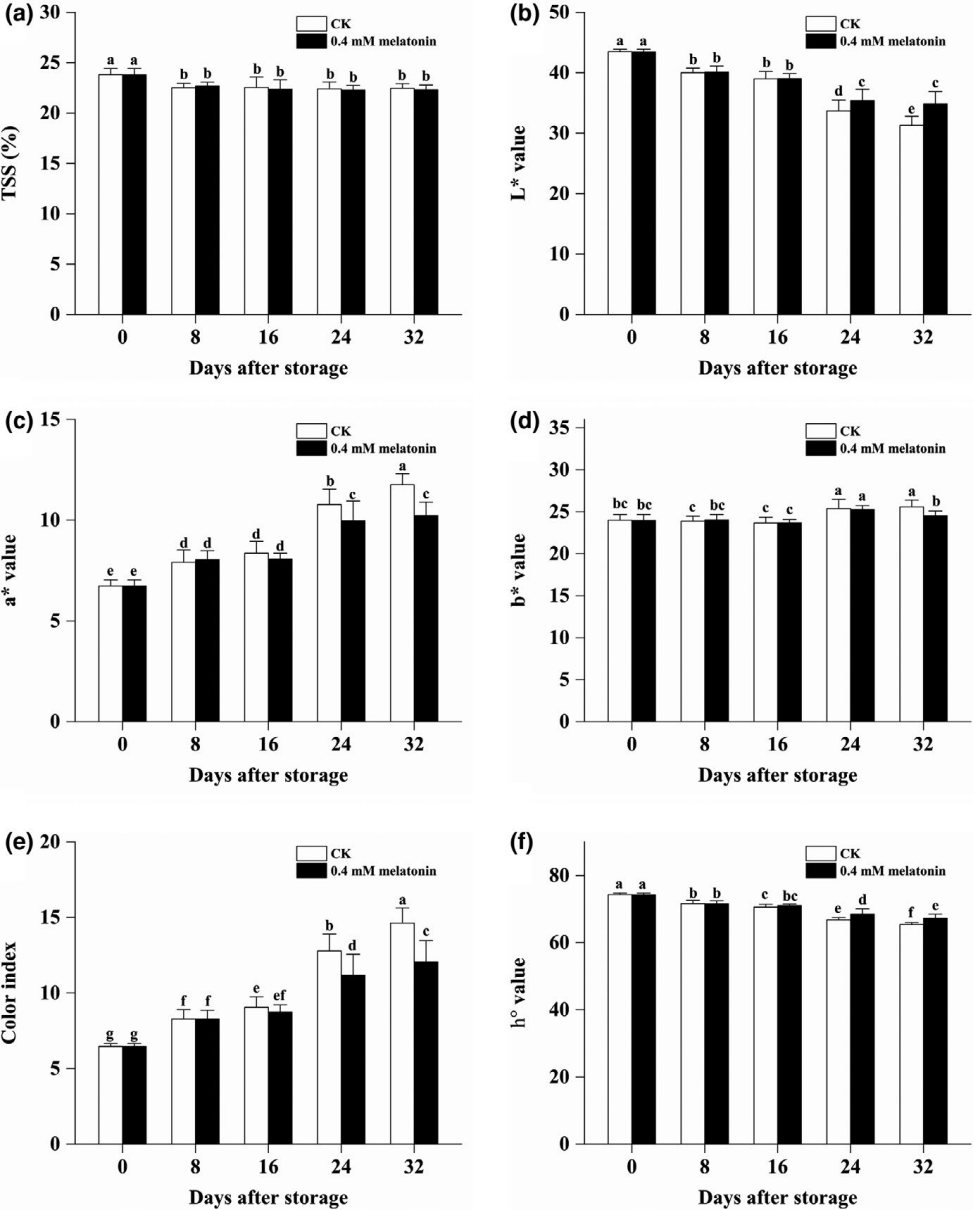
Changes in TSS content and chromatic values of CK and MLT‐treated longan fruits during low‐temperature storage. (a) TSS contents, (b) L* value, (c) a* value, (d) b* value, (e) color index, and (f) h* value (hue angle) of “Chuliang” longan fruits during the low temperature (4 ± 1°C) storage. Note: different lowercase letters indicated a significant difference, *p* < .05

The TSS contents in both of the CK and MLT‐treated longan fruits were found to be quickly decreased (from 23.80% to 22.71%) in the first 8 days and then slowly decreased to be about 22.36% at 16 DAS and showed a stable level in the next 24 days. However, no significant difference of TSS content was found between CK and MLT‐treated longan fruits at each time point (Figure 1a). Significantly higher chromatic L* and h° value but lower chromatic a*, b* value and CI value were observed on the MLT‐treated longan pericarp at the two storage stages (Figure 1b–f). The above results indicated that the MLT treatment did not significantly influenced the TSS content, but inhibited the deterioration of longan fruits’ appearance at the later stages of low‐temperature storage.

### 3.2 Effect of MLT treatment on the browning, relative electrolytic leakage, and MDA content in longan pericarp

**FIGURE 2:**
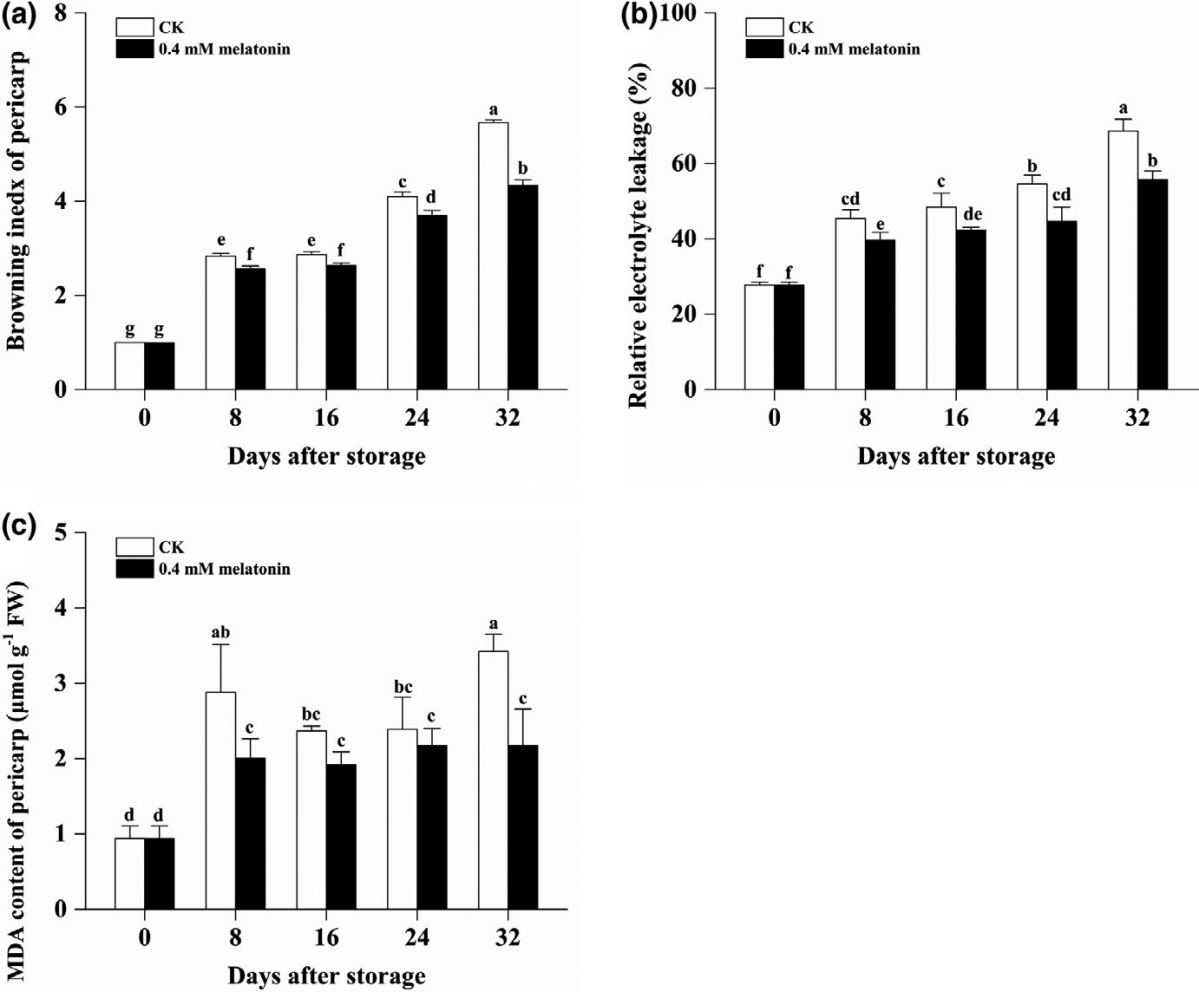
The pericarp browning index, relative electrolytic leakage, and MDA content of the control and MLT‐treated longan fruits during low‐temperature storage. Note: different lowercase letters indicated a significant difference, *p* < .05

As shown in Figure 2a, lower browning index was observed on the MLT‐treated longan pericarp during the whole storage (from 0 DAS to 32 DAS). In details, pericarp browning index of CK longan was increased from 2.83 to 5.67, while pericarp browning index of MLT‐treated longan was increased from 2.57 to 4.33 during the storage. Accordingly, lower relative electrolytic leakage of pericarp was observed in the MLT‐treated longan pericarp during the whole storage (from 0 DAS to 32 DAS). The relative electrolytic leakage of CK pericarp increased from 27.78% to 68.61% while that of the MLT‐treated longan pericarp slowly increased from 27.78% to 55.71% (Figure 2b). The MDA content of CK pericarp increased quickly from 0.94 to 2.88 μmol/g FW during the first 8 days and then increased to be 3.42 μmol/g FW at 32 DAS but the MDA content of MLT‐treated longan pericarp was only 2.01 μmol/g FW at 8 DAS and 2.18 μmol/g FW at 32 DAS (Figure 2c). These results suggested that MLT significantly inhibited the pericarp browning and effectively maintained the integrity of pericarp cell membranes.

### 3.3 Effect of MLT treatment on the PPO enzymatic activity, superoxide anion, and H_2_O_2_ content in longan pericarp

**FIGURE 3:**
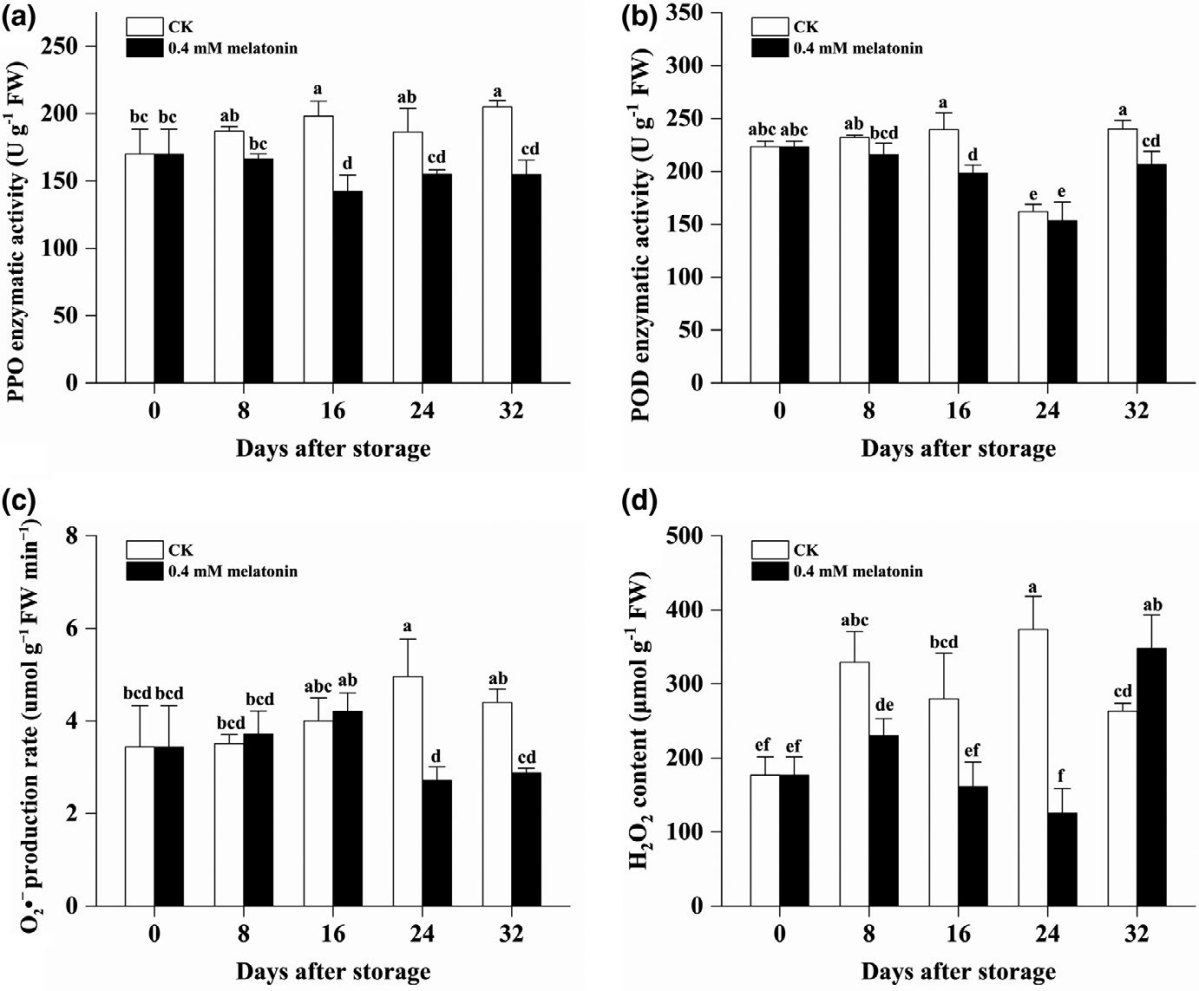
Polyphenol oxidase enzymatic activity (a), POD enzymatic activity (b), production rate of O2 ^•−^ (c), and H2O2 content (d) in the pericarp of the CK and MLT‐treated longan fruits during low‐temperature storage. Note: different lowercase letters indicated a significant difference, *p* < .05

As shown in Figure 3a, the enzymatic activity of PPO in the CK pericarp was observed to be significantly higher than that of PPO in the MLT‐treated longan pericarp from 16 DAS to 32 DAS. In details, the enzymatic activity of PPO in the CK longan pericarp increased from 169.92 to 205.01 U g^‐1^ FW during the whole storage, while that of PPO in the MLT‐treated longan pericarp decreased from 169.92 to 142.187 U g^‐1^ FW from 0 DAS to 32 DAS (Figure 3a). In total, the POD activity in the MLT‐treated longan pericarp was lower than that in the CK pericarp during the whole storage although the POD activity in the MLT‐treated longan pericarp was only found to be significantly lower at 16 and 32 DAS (Figure 3b).

The content of two oxygen radical species was also determined. Significantly lower O_2_^•−^ production rate was observed in the MLT‐treated longan pericarp at the last two stages (Figure 3c). In details, the O_2_^•−^ production rate in the CK longan pericarp increased from 3.44 to 4.96 μmol/g FW min^‐1^ from 0 DAS to 24 DAS and then showed a nonsignificant decrease, while the O_2_^•−^ production rate in the MLT‐treated longan pericarp increased nonsignificantly from 3.44 to 4.21 μmol/g FW min^‐1^ from 0 DAS to 16 DAS, and then showed a significant decrease at the last two stages (24 DAS: 2.71 μmol/g FW min^‐1^; 2.88 μmol/g FW min^‐1^; nonsignificant between them) (Figure 3c). Moreover, significantly lower H_2_O_2_ content was observed in the MLT‐treated longan pericarp at 8 DAS, 16 DAS, and 24 DAS (Figure 3d). In details, the H_2_O_2_ content in the CK longan pericarp quickly increased from 176.69 μmol/g FW to 329.40 μmol/g FW from 0 DAS to 8 DAS and continuously increased to be 373.53 μmol/g FW at 24 DAS and then decreased to 263.21 μmol/g FW at 32 DAS. The H_2_O_2_ content in the MLT‐treated longan pericarp increased from 176.69 to 230.41 μmol/g FW at the first 8 days but then decreased to 125.47 μmol/g FW at 24 DAS. The above results indicated that the MLT treatment was effective to inhibit the increase in PPO enzymatic activity and reduce POD activity, O_2_^•−^ production rate and H_2_O_2_ content in longan pericarp during the low‐temperature storage.

### 3.4 Effect of MLT treatment on the contents of ascorbic acid, glutathione, flavonoid, and phenolics in longan pericarp

**FIGURE 4:**
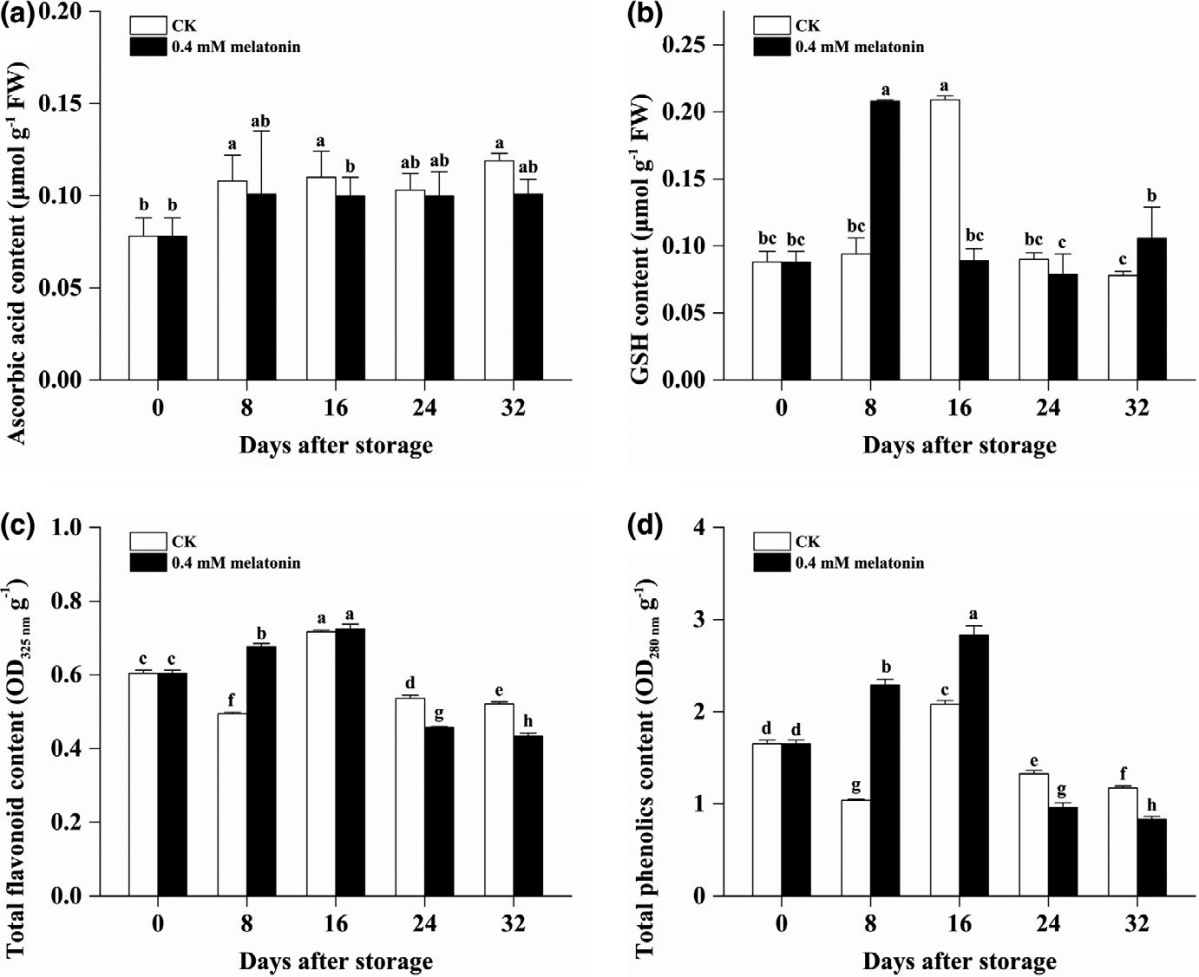
The content of ascorbic acid (a), GSH (b), total flavonoid (c), and total phenolics (d) in the pericarp of the CK and MLT‐treated longan fruits during low‐temperature storage. Note: different lowercase letters indicated a significant difference, *p* < .05

No significant difference of ascorbic acid content was found between the CK and the MLT‐treated longan pericarp (except 16 DAS) (Figure 4a). Higher GSH content was, respectively, found at 8 DAS and 32 DAS but lower GSH content was found at 16 DAS in the MLT‐treated longan pericarp (Figure 4b). Moreover, higher total flavonoid content was detected at 8 DAS (Figure 4c) and higher total phenolics content was observed at 8 DAS and 16 DAS in the MLT‐treated longan pericarp during the low‐temperature storage (Figure 4d). It was unexpected that lower total flavonoid content and lower total phenolics content were found in the MLT‐treated longan pericarp at 24 DAS and 32 DAS (Figure 4c–d). The above results illustrated that the MLT treatment might promote the accumulation of antioxidants in longan pericarp at the earlier stages of storage.

### 3.5 Effect of MLT treatment on activities of SOD, APX, and CAT in longan pericarp

**FIGURE 5:**
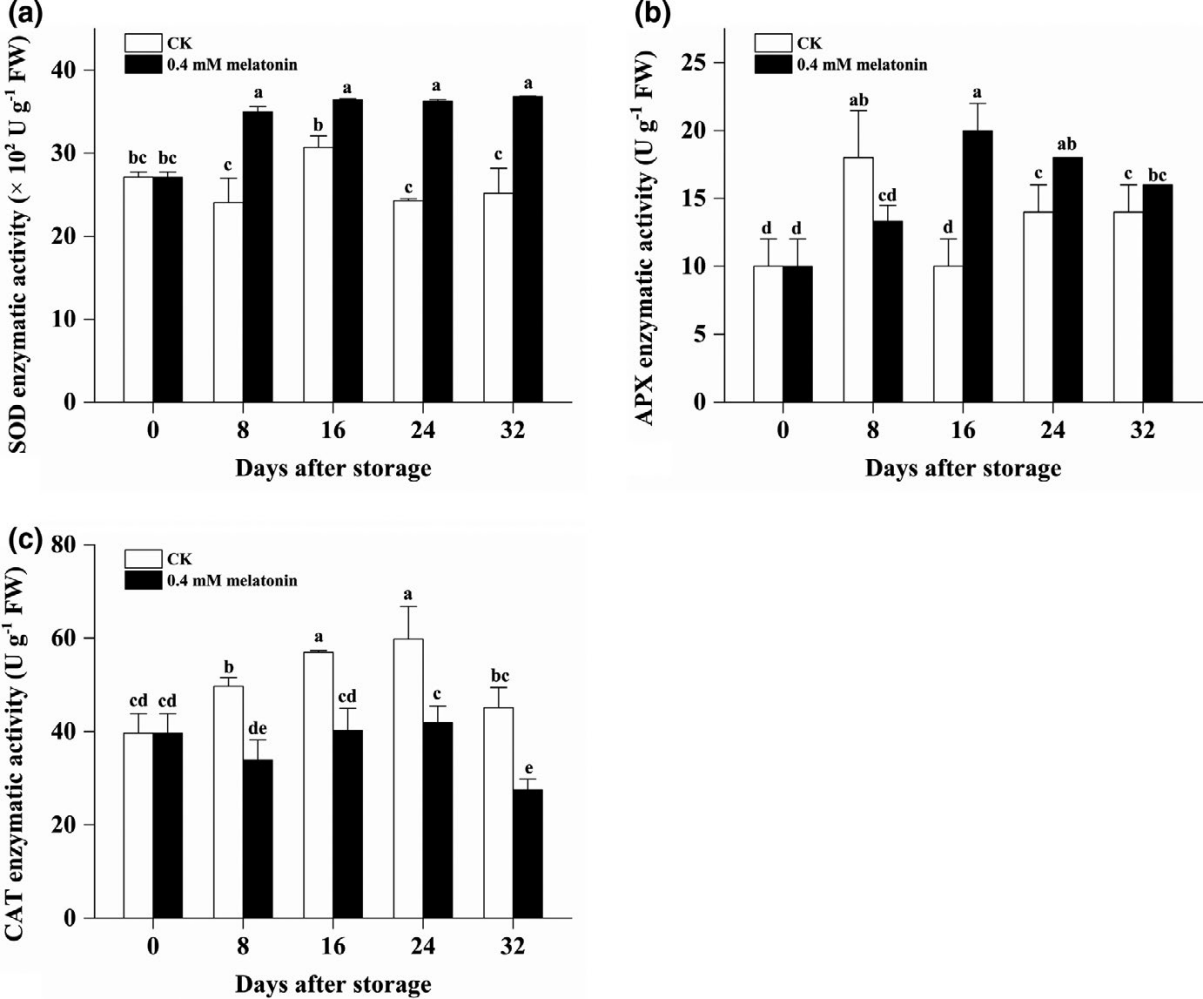
The enzyme activity of SOD (a), APX (b), and CAT (c) in the pericarp of the CK and MLT‐treated longan fruits during low‐temperature storage. Note: different lowercase letters indicated a significant difference, *p* < .05

The SOD activity in the MLT‐treated longan pericarp quickly decreased from 2,710.37 to 3,500.84 U g^‐1^ at 8 DAS and then kept at high level at the later three stages, which was parallelly higher than that in the CK pericarp during the whole storage (Figure 5a). Similarly, the APX activity in the MLT‐treated longan pericarp increased from 10.00 to 20.00 U g^‐1^ from 0 DAS to 16 DAS and then slowly decreased to be 16.00 U g^‐1^, which was found to be lower at 8 DAS but higher at 16 DAS and 24 DAS than the APX activity in the CK pericarp (Figure 5b). It was worthy to note that the CAT activity in the MLT‐treated pericarp were lower than that in the CK pericarp at each time from 8 DAS to 32 DAS (Figure 5c). The CAT activity in the CK pericarp increased from 39.67 to 59.80 U g^‐1^ during the first 24 days and then decreased to be the initial level. The CAT activity in the MLT‐treated pericarp did not increase during the first 24 days and then significantly decreased from 41.93 to 27.53 U g^‐1^. These results suggested that MLT might induce some enzymatic activities (i.e., SOD and APX) for scavenging free radical while reduce the induction of another enzymatic activities for scavenging free radical (i.e., CAT and POD) due to its direct scavenging on free radicals.

## 4 DISCUSSION

### 4.1 Effect of MLT treatment on the quality and postharvest enzymatic pericarp browning of longan fruits

Postharvest application of MLT had been reported to effectively improve the storage performance in many fruits. Onik et al. reported that 1 mM MLT treatment significantly reduced the weight loss and MDA content, maintained better skin structure of apple during storage at 1°C for 56 days (Onik et al., 2020). Wang et al. found that immersing in 200 μM MLT significantly reduced berry abscission and rotten index of table grape stored at 4 ± 0.5°C (Wang et al., 2020). Hu et al. (2018) reported that 100 μM MLT lead to delayed decrease of firmness, starch contents and color L* value, as well as lower mass loss rate and decay incidence in kiwifruit. MLT application was also reported to do not influence the TSS content, titratable acidity and color factors of mango fruits, but 1,000 μM MLT was able to reserve the firmness of mango during storage (Rastegar et al., 2020). Gao et al. (2016) found that 100 μM MLT treatment for 10 min reduced weight loss and decay incidence, maintained firmness and TSS content, significantly decreased the MDA content in peach fruits stored at 25–28°C. Treatment with 100 μM MLT was found to reduce rates of respiration, inhibit the softening and ripening, and delay the senescence of European pear fruit (Liu et al., 2019). Application of 0.1 or 1 mmol/L MLT on strawberry fruit was reported to effectively reduce decay and weight loss, delay senescence by maintaining the color, firmness, TSS content, and titratable acidity (Liu et al., 2018). Dipping in 0.1 mM MLT for 60 min significantly inhibited the gray mold development caused by *Botrytis cinerea* in cherry tomato stored at 22 ± 1°C (Li et al., 2019). Moreover, 0.4 mM MLT strongly suppressed pericarp browning and delayed discoloration by declining loss of total phenolics, flavonoids, and anthocyanins of litchi pericarp (Zhang et al., 2018). Treatment with 100 μM MLT resulted in increased chilling tolerance in pomegranate fruit (stored at 4°C) disclosed as lower husk browning accompanying by higher membrane integrity and lower electrolyte leakage and MDA accumulation (Jannatizadeh et al., 2019). Therefore, MLT effectively maintains postharvest quality and delays senescence of fruits by affecting physiological state, maintaining fruit external color and hardness, reducing respiration rate, membrane permeability and mass loss, inhibiting ripening, chilling injury, berry abscission, disorder and so on.

In this study, postharvest MLT treatment did not significantly influence the TSS content of longan fruits, but helped maintain a better appearance quality by delaying the decrease of L* value (lightness) and h° value and inhibiting the increase of a* value (redder), b* value (more blue), and CI index of pericarp. Consistent with the results of chromatic values, the increase in pericarp browning was significantly suppressed in treated longan fruits throughout the low‐temperature storage. This results might be related to the effect of MLT on delaying increase in relative electrolytic leakage and MDA content in longan pericarp, which is beneficial to the occurrence of enzymatic browning by PPO and POD. The enzymatic browning was effectively inhibited in the MLT‐treated longan pericarp, which was disclosed by the lower PPO and POD activities throughout the low‐temperature storage. More importantly, lower relative electrolytic leakage and MDA content in the MLT‐treated longan pericarp indicated a protecting effect of MLT on cytomembrane integrity. Thus, 0.4 mM MLT was proved to be a useful treatment to delay pericarp browning and maintain the external quality of longan fruits by protecting cytomembrane integrity of pericarp under low‐temperature storage.

### 4.2 Effect of MLT treatment on the antioxidants and nonenzymatic scavenging of ROS

The imbalance between scavenging ability and production of oxygen free radicals might lead to the burst of ROS (Lin et al., 2014), hydrogen peroxide‐induced energy deficiency (Lin, Lin, Lin, et al., 2016), and peroxidation of membrane lipid (Lin, Lin, Chen, et al., 2016) and further accelerate fruit senescence and browning caused by PPO and POD in longan fruit (Jiang et al., 2002). Wang et al. (2020) reported that 200 μM MLT immersion greatly enhanced amino acids accumulation and significantly increased phenolics content of “Kyoho” grape stored at 4 ± 0.5°C. Hu et al. (2018) reported that 100 μM MLT significantly increased the contents of ascorbic acid and glutathione in kiwifruit stored at 0 ± 0.5°C. Rastegar et al. (2020) found that 1 mM MLT application significantly inhibited the decrease in phenolics, flavonoids, and antioxidants of mango during storage at 15 ± 1°C. In peach fruits stored at 25–28°C, 100 μM MLT treatment for 10 min was observed to maintain ascorbic acid and reduce the production rate of O_2_•^−^ and H_2_O_2_ content (Gao et al., 2016). Application of 0.1 or 1 mmol/L MLT was notably effective to increase the total phenolics and flavonoid contents, which resulted in the higher antioxidant capacity in strawberry fruit but had a negative impact on the ascorbic acid content (Liu et al., 2018). Dipping in 0.1 mM MLT for 60 min significantly lead to higher total phenolics, flavonoids, and lignin content in cherry tomato stored at 22 ± 1°C (Li et al., 2019). Treatment with 0.4 mM MLT strongly delayed loss of total phenolics, flavonoids, and anthocyanins and inhibited the generation of superoxide radicals (O_2_·−), hydrogen peroxide (H_2_O_2_), and MDA of litchi pericarp during storage at 25°C for 8 days (Zhang et al., 2018). Treatment with 100 μM MLT resulted in increased phenols accumulation and lower ROS (H_2_O_2_) content in pomegranate fruit stored at 4°C (Jannatizadeh et al., 2019). The above documents indicated that nonenzymatic scavenging of ROS through higher antioxidants was induced by MLT treatment with an appropriate concentration in many fruits. In this study, 0.4 mM MLT treatment significantly inhibited the increase of production rate of O_2_^•−^ and H_2_O_2_ content. This might be related to the promoting effect of MLT treatment on the accumulation of glutathione and flavonoids especially of phenolics at earlier storage stages in longan pericarp. It was worthy to note that the treatment have no positive impact but a little negative effect on the content of ascorbic acid. In total, 0.4 mM MLT was an effective treatment on inhibiting longan pericarp browning due to the nonenzymatic scavenging of oxygen free radicals by increasing the accumulation of antioxidants (mainly glutathione, flavonoids, and phenolics) during the low‐temperature storage.

### 4.3 Effect of MLT treatment on the enzymatic scavenging of oxygen free radicals

Superoxide dismutase, CAT, POD, and APX are the key antioxidant enzymes for scavenging ROS in plant. Among them, SOD can protect cell from oxidant stress by dismutating O_2_^•−^ to H_2_O_2_, while CAT, POD, and APX sequentially degrade H_2_O_2_ to H_2_O and O_2_ (Gao et al., 2016). Hu et al., reported that 100 μM MLT maintained higher SOD, APX, and glutathione reductase (GR) activity, increased the peak of catalase in kiwifruit stored at 0 ± 0.5°C (Hu et al., 2018). Rastegar et al., found that 1 mM MLT application significantly increased the activity of the CAT and POD enzymes in mango during storage at 15 ± 1°C (Rastegar et al., 2020). In peach fruits stored at 25–28°C, 100 μM MLT treatment for 10 min was proved to significantly enhanced the activities of SOD, CAT, POD, and APX in both cultivars (“Shahong” and “Qinmi”) (Gao et al., 2016). Jannatizadeh reported that treatment with 100 μM MLT resulted in higher activities of ROS scavenging enzymes (CAT, SOD, APX, and GR) in pomegranate fruit stored at 4°C (Jannatizadeh et al., 2019). Treatment with 0.4 mM MLT significantly enhanced the activities of antioxidant enzymes including SOD, CAT, APX, and GR in litchi pericarp during storage at 25°C for 8 days (Zhang et al., 2018). Consistent with the above reports, our results indicated that 0.4 mM MLT significantly enhanced the activities of SOD and APX. However, the MLT treatment had a negative impact on activities of CAT and POD. This result might be explained that the induction of CAT activity from ROS was eliminated by the scavenging effect from increased flavonoid and phenolic content as well as enhanced SOD and APX activities. POD was regarded to be acted together with PPO enzyme to promote pericarp browning in litchi and longan. Consistent with the result in litchi pericarp, POD and PPO were also observed to be inhibited by MLT treatment in this study. These results indicated that 0.4 mM MLT help longan fruits prevent pericarp browning by significantly enhancing the activities of antioxidant enzymes SOD and APX.

## 5 CONCLUSION

Postharvest 0.4 mM MLT treatment did not significantly influence the quality of longan fruits, but it effectively inhibited the increase in membrane permeability, O_2_^•−^, and H_2_O_2_ content, as well as activities of browning‐related enzymes PPO and POD, which was the main cause of pericarp browning. More importantly, the MLT treatment promoted the increase in antioxidants such as GSH, flavonoid, and phenolic acid at earlier storage stages and enhanced the activities of enzymes for scavenging ROS (SOD and APX). Thus, 0.4 mM MLT treatment effectively blocked the development of postharvest pericarp browning and improved the exterior quality of “Chuliang” longan fruits. However, further investigation is still essential to explore the molecular basis for the appearance improving effect of MLT on longan fruits.

## CONFLICT OF INTEREST

The authors declare no conflict of interest.

## ETHICAL APPROVAL

The study does not involve any human or animal testing.

## DATA AVAILABILITY STATEMENT

The data that support the findings of this study are available from the corresponding author, upon reasonable request.
